# High Injury Incidence Among Youth in the World's Largest Handball Tournament: A Prospective Observational Study of 17 034 Participants

**DOI:** 10.1111/sms.70285

**Published:** 2026-04-11

**Authors:** Ida Lindman, Josefin Abrahamson

**Affiliations:** ^1^ General Practice/Family Medicine, School of Public Health and Community Medicine, Institute of Medicine, Sahlgrenska Academy University of Gothenburg Gothenburg Sweden; ^2^ Research, Education, Development & Innovation Primary Health Care Sweden; ^3^ Department of Health and Rehabilitation Institute of Neuroscience and Physiology at Sahlgrenska Academy, University of Gothenburg Sweden; ^4^ SportsMed AB Gothenburg Sweden

**Keywords:** adolescence, handball injuries, injury incidence, sports injuries, youth handball

## Abstract

Partille Cup is the world's largest youth handball tournament. Research among youth handball players, and especially during tournaments, is scarce. The primary aim of this prospective study was to examine the incidence of injuries among youth handball players in Partille Cup. The secondary aim was to compare injury incidence by age, sex, and across days of the tournament. All visits at medical tents were recorded. According to FIFA's suggestion, an injury was defined as any physical condition that prompted a handball player to seek medical care during the tournament. In 2024, 17 034 players participated in the tournament over six consecutive days. A total of 1072 injuries were reported, resulting in an overall injury prevalence of 6.3% (95% CI 5.9%–6.7%) and an injury incidence of 34.9 injuries/1000 player hours (95% CI 32.8–37.0), with no sex differences. Injury incidence increased progressively throughout the tournament, with the lowest rate on the first day (21.4 injuries/1000 player hours, 95% CI 0.4–41.4) and highest on day 6 (46.6 injuries/1000 player hours, 95% CI 34.1–59.0). Injury incidence varied significantly across age groups, with the youngest players (aged 10 and 11 years) exhibiting the lowest injury rates (13.9, 95% CI 7.1–20.6 and 13.4, 95% CI 7.8–19.0, injuries/1000 player hours, respectively). Lower extremity injuries were most common (30%), closely followed by wound injuries (29%) and upper extremity injuries (28%). These findings illustrated the injury patterns observed during a large, six‐day youth handball tournament and offer important insights for shaping medical strategies in future handball tournaments.

## 1 Background

Handball is a popular team sport in Europe and is played by millions of individuals worldwide [1, 2, 3]. Participation in physical activity such as handball offers numerous health benefits, including improved cardiovascular fitness, muscular strength, coordination, and social development [4, 5, 6, 7].

Although youth handball is widely regarded as beneficial, players in this age group remain vulnerable to injuries [3, 8, 9, 10]. Handball is a fast‐paced contact sport characterized by repeated jumping and landing, rapid accelerations and decelerations, frequent changes in direction, and nonetheless, physical contact, all of which increase the risk of injury [11]. It is one of the sports with the highest injury rates (82%) at the Olympic games [12]. Both lower and upper extremities are common injury sites in handball. The knee and ankle are particularly vulnerable in the lower body, while the shoulder, wrist, and fingers are frequent upper‐body injury sites due to repeated ball handling, blocking, and physical contact [13, 14, 15, 16].

Previous studies on youth handball players have reported overall injury incidences ranging between 1.0 and 26.0 injuries per 1000 h of play [8, 10, 14, 16, 17, 18]. Higher injury incidence has been seen during games than training, with up to 33 injuries per exposure hour among youth [2, 8, 16, 18]. While injury epidemiology in adult and elite handball has been extensively studied, considerably less is known about injury incidence and characteristics among younger players, particularly those aged 10–13 years and female adolescents. Moreover, most studies have been conducted during full seasons, and few have investigated injuries during tournament play [17].

Injury prevention in handball has advanced in recent years, particularly through structured warm‐up and neuromuscular training programs [19, 20, 21, 22]. However, tournaments pose unique challenges due to match congestion and limited recovery time, especially for youth players. Describing injury incidence and characteristics during large youth handball tournaments may contribute to a better understanding of injury patterns in this specific context and help inform future research, medical preparedness, and the development of context‐specific preventive approaches.

Since 1970, Gothenburg in Sweden has hosted Partille Cup, the largest international youth handball tournament in the world. The 2024 edition featured 1306 teams from 38 nations, playing more than 4000 matches over six days [23]. Using medical records from this tournament, this study aimed to investigate the incidence and pattern of injuries among youth handball players participating in Partille Cup 2024. The secondary aim was to compare injury incidence between age groups, sex, and across the duration of the tournament.

## 2 Method

### 2.1 Study Design

This study was conducted as a prospective observational study. The study followed the reporting guidelines according to the STROBE extension: the STROBE Sports Injury and Illness Surveillance (STROBE‐SIIS) [24].

### 2.2 Ethics

This study was approved by the Swedish Ethical Review authority with diary number 2023‐06647‐01. All data were gathered anonymously, without any personal identifiers recorded, ensuring participant confidentiality and privacy. For all minor participants, a responsible adult (parent, legal guardian, or team representative) approved medical assessment and treatment during the tournament. Athletes and guardians, when relevant, were informed about the anonymously collected data. The study was conducted in accordance with the principles of the Declaration of Helsinki.

### 2.3 Study Participants

Inclusion criteria were handball players aged 10–21 years old participating at Partille cup during the tournament in 2024. No exclusion criteria were applied. During the tournament, multiple on‐site medical tents were established, which were easily accessible and free of charge to all players. The tents were staffed by trained first‐aid personnel including nurses, nurse assistants, and physiotherapists. All players seeking medical care were recorded to count for injuries.

### 2.4 Data Collection

All medical tent‐visits were recorded in a custom tournament‐specific application. Each patient chart included the player's age, sex, nationality, injury location, treatment, and outcome (e.g., discharged, transported for further physical examination, or ambulance arrival). Contacts unrelated to handball injuries or illnesses were documented yet excluded from the analysis. This method of recording injuries has been used in previous tournament injury studies [25].

### 2.5 Definition of Injury

An injury was defined according to FIFA as “any physical condition” that led a handball player to seek medical attention during the tournament. This definition aligns with previous studies on the same topic and is widely used worldwide [14, 25]. Although the definition is originally stated by FIFA, it has been used for several sports and is not unique for soccer. Using this definition provides future tournament organizers with essential guidance for appropriately planning medical support.

### 2.6 The Tournament

The tournament is played according to the rules of the International Handball Federation and the Swedish Handball Federation. Teams are divided into age categories for boys and girls separately. The competition begins with a group stage (each team in a group plays all other teams) followed by a playoff‐knockout stage. The age group 8–9 is called kinder‐play and has no playoffs and was excluded from this study. In the 10–15‐year age categories, the two best teams from each group advance to the A‐playoffs, the next two to the B‐playoffs, and the remaining teams to a C‐playoff. The minimum number of played games for each team during the tournament is six. Match durations are generally 2 × 15 min for all age groups, with the older age categories (18–21 years) playing 2 × 20 min in playoff matches [23]. The games are played over six consecutive days; however, the first day is dedicated solely to kinder‐play, while the special group, “European Open,” kicks off its competition with 20 games on Monday. European Open is a tournament for some of the world's best youth national teams. All other groups begin their competition on day two.

### 2.7 Statistics

For injury prevalence and incidence, the corresponding 95% confidence interval (CI) was calculated according to Knowles et al. [26]. Player hours were calculated as the number of games in each age group multiplied by the corresponding game time multiplied by the number of players on the field in each game. The Chi^2^ test was used for comparisons between categorical variables, and the independent *t*‐test was used for comparisons of continuous variables. For comparisons of injury incidence between groups (sex, age groups, and day of the tournament), injury incidence rate ratios (IRRs) were calculated. The IRRs were calculated, in accordance with Knowles et al. [26], as the ratio of two incidence rates (injuries/1000 player hours). Age groups were categorized according to the divisions used in the tournament. Statistical significance was considered present if the 95% CI between two groups did not overlap or had a *p*‐value < 0.05. For the IRRs, the difference in injury incidence was considered statistically significant if the 95% CI did not include 1.00. Differences in injury localization between sexes were analyzed using the Chi^2^ test. When a significant association was observed, cell contributions were examined using adjusted standardized residuals. Effect size was estimated using Cramér's V. The data were analyzed using IBM SPSS Statistics for Mac, version 29.0 (IBM Corp., Armonk, NY) and Microsoft Excel.

## 3 Results

A total of 4295 games were played by 17 034 players (52% girls) (Table 1). The total number of reported injuries during the tournament was 1072, of which 575 (54%) were among girls, 495 (46%) among boys, and two (0.2%) with unreported sex. Mean age at the time of reporting an injury was 14.9 (SD 2.0) years, where girls were significantly younger (mean 14.6, SD 1.9) compared with boys (mean 15.2, SD 2.1, *p* < 0.001). Most injured players were from the Nordic countries (68%), of which Sweden accounted for half of this (34%). Twenty‐two percent were from the rest of Europe, 5% from South America, 3% from Asia, 2% from Africa, and 0.4% from North America.

**TABLE 1:** Demographics and injury data in total and stratified by sex.

	Total	Girls	Boys
Games, *n*	4295	2253	2042
Total number of players, *n* (%)	17 034^a^	8858 (52%)	8176 (48%)
Total playing hours, (mean hours/player)	30 758 (1.80)	16 205 (1.83)	14 497 (1.77)
Number of injuries, *n* (%)	1072 (100%)	575 (53.6%)	495 (46.2%)
Mean age at injury, years (SD)	14.9 (2.0)	14.6 (1.9)	15.2 (2.1)^b^
Injury prevalence, % (95% CI)	6.3 (5.9–6.7)	6.5 (6.0–7.0)	6.1 (5.5–6.6)
Injuries/1000 player hours, (95% CI)	34.9 (32.8–37.0)	35.5 (32.6–38.4)	34.1 (31.1–37.2)

Abbreviations: CI, confidence interval; h, hours; *n*, number; SD, Standard deviation.

^a^ 2 players had unreported sex.

^b^ Significant difference between sex, *p* = < 0.001.

### 3.1 Injury Prevalence

Table 1 shows demographics and injury data. The injury prevalence for the tournament in total was 6.3% (95% CI 5.9%–6.7%), with no significant difference between girls (6.5%, 95% CI 6.0%–7.0%) and boys (6.1%, 95% CI 5.5%–6.5%) (*p* = 0.2). See Table S3. There were in total 34.9 (95% CI 32.8–37.0) injuries/1000 player hours, with no significant difference between girls (35.5, 95% CI 32.6–38.4) and boys (34.1, 95% CI 31.1–37.2) (IRR 0.96, 95% CI 0.85–1.09) (Table 1). The total number of playing hours was 30 758 (1.8 h per player).

### 3.2 Injury Incidence Throughout the Tournament

**FIGURE 1:**
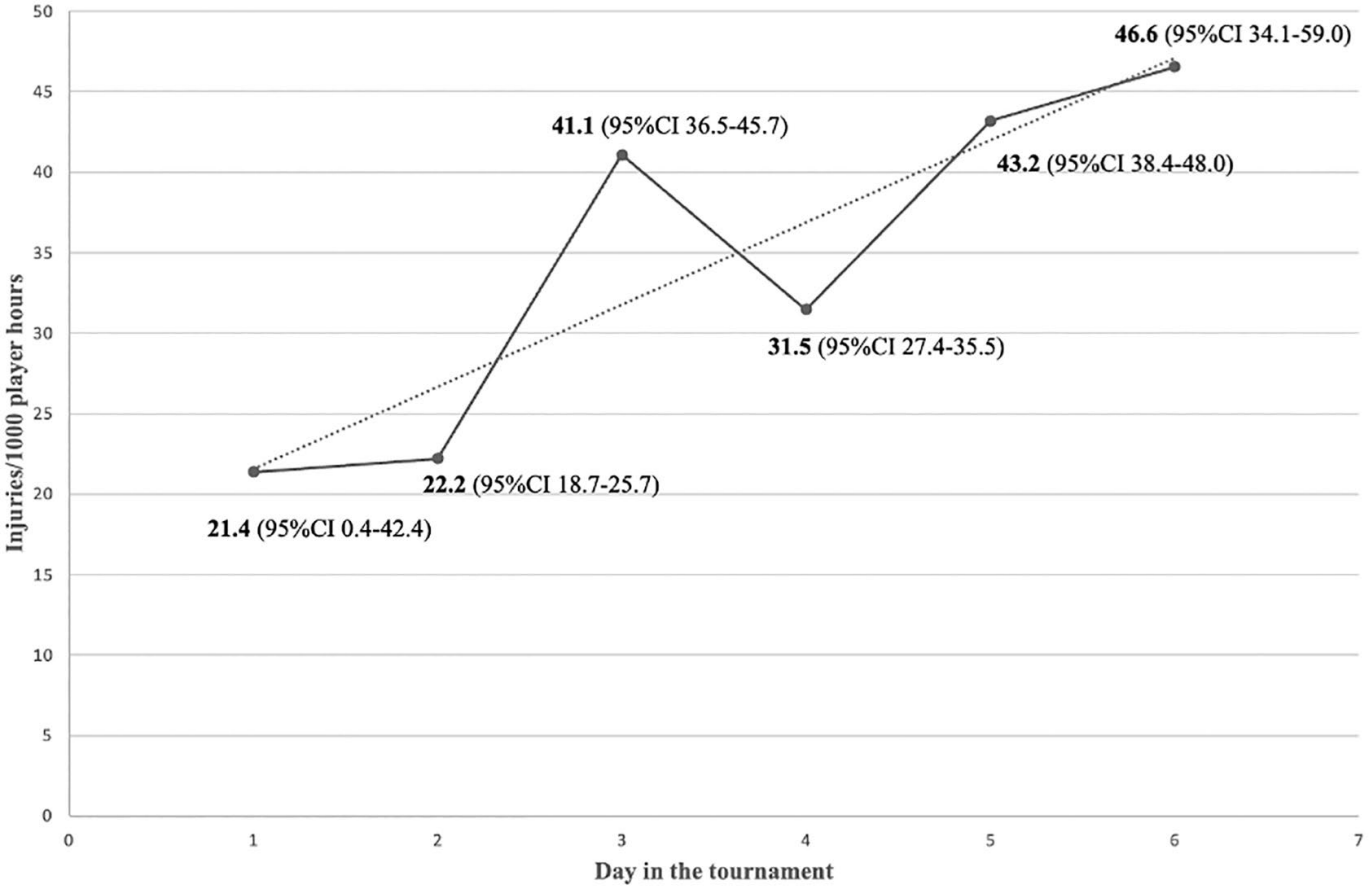
Injury incidence defined as injuries/1000 player hours between the different days in the tournament. The incidence differed significantly between day 2 and 3, 4, 5, 6 and between day 3 and 4 of the tournament. Plotted line = linear trend line.

The injury incidence increased as the tournament went on, with the lowest injury incidence on day 1 (21.4 injuries/1000 player hours, 95% CI 0.4–42.4) and the highest on the last day, day 6 (46.6 injuries/1000 player hours, 95% CI 34.1–59.0) (Figure 1). However, although the IRR for injury incidence between day 1 and day 6 was the highest at 2.2 (95% CI 0.79–6.01), this was not statistically significant as shown by the 95% CI. The injury incidence differed significantly between day 2 and all other days of the tournament, except for day 1, with the highest IRR between day 2 and day 6 (2.1, 95% CI 1.5–2.9). There was also a significant difference between day 3 and day 4 with an IRR of 0.77 (95% CI 0.65–0.91). There was also a clear increase in injury incidence on day 3, which reached levels comparable to the peak observed on day 6, highlighting an early mid‐tournament surge. See Table S2 for additional IRRs with 95% CI between all days in the tournament.

### 3.3 Severity of Injury

Of the registered injuries, 0.6% (*n* = 6) were so severe that an ambulance was required, and in 4% (*n* = 40) of the cases, the players were transported independently or with the help of medical transport to a physician for further evaluation. Head injuries accounted for 50% (*n* = 3) of the injuries where an ambulance was required. However, upper extremity injuries (*n* = 21, 52.5%) were most common in athletes needing transportation for further evaluation. All six injuries requiring an ambulance and half of the injuries requiring other transportation for further evaluation occurred during day 3 of the tournament.

### 3.4 Injuries Across Age Groups

**FIGURE 2:**
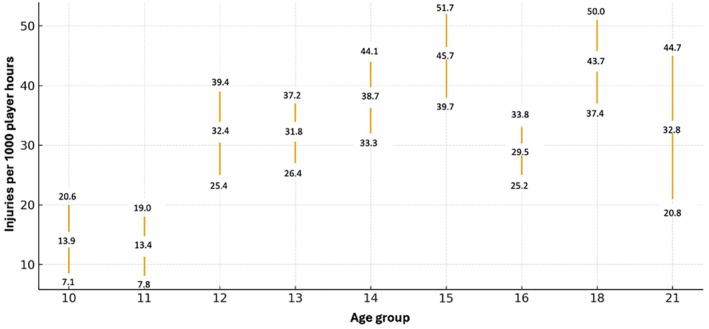
Injury incidence stratified by age group (10–21 years). The middle represents the observed injuries per 1000 player hours for each age category; error bars indicate 95% confidence interval. Age categories are plotted with equal horizontal spacing for clarity.

Figure 2 shows injuries/1000 player hours between the different age groups. See Table S1 for all IRRs with 95% CI between all age groups where each age group is analyzed separately. The youngest age groups (10 and 11 years) had a significantly lower incidence of injuries (13.9, 95% CI 7.1–20.6, and 13.4, 7.8–19.0 injuries/1000 player hours respectively), compared with the other age groups (29.5–45.7, 95% CI 25.2–51.7, injuries/1000 player hours). The IRRs between the youngest age groups (10 and 11 years) and the other age groups (12–21 years) were between 2.1–3.4 (95% CI 1.3–5.5). The 15‐year old age group had the highest injury incidence (45.7, 95% CI 39.7–51.7 injuries/1000 player hours) which differed significantly compared with the age groups 10, 11, 12, 13 and 16.

### 3.5 Injury Location

**FIGURE 3:**
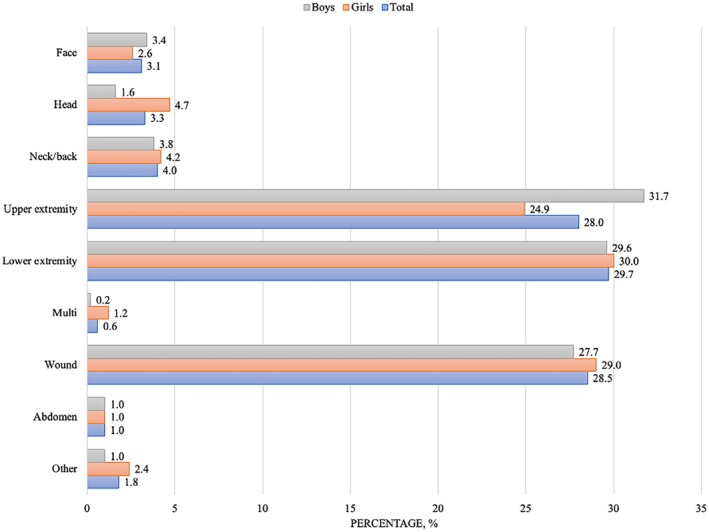
Injury location in total and stratified by sex. Gray bar = boys; red bar = girls and blue bar = total.

Figure 3 shows the distribution of injury localization in total and stratified by girls and boys. The most common injury locations were injuries to the lower extremity (30%), wound injuries (29%), and injuries to the upper extremity (28%). The distribution of injury localization differed significantly between the sex (*p* = 0.011). However, the effect size was very small (Cramér's V = 0.011), indicating a weak association between sex and injury localization.

Lower extremity injuries were most common in girls (30%) and upper extremity injuries were most common in boys (32%). Examination of adjusted standardized residuals showed that head injuries were more frequent among girls than expected, while injuries to the upper extremities were more common among boys. No other injury locations demonstrated statistically meaningful deviations from expected frequencies (see Table S4 for all adjusted standardized residuals).

## 4 Discussion

The most important finding of this prospective study among youth handball players during a tournament is the high incidence of 34.9 injuries per 1000 player hours, with the lowest injury rates among the youngest (10–11 years old) and increased injury rates throughout the tournament. There are several previous studies on handball; however, this is one of the few studies considering youth handball tournaments including 17 034 players and 4295 games during one tournament over six consecutive days.

In this study, the injury incidence was 34.9 injuries per 1000 player hours (95% CI 32.8–37.0), with no significant differences between girls and boys. The high injury incidence aligns with previous studies showing that handball carries a relatively high risk of injury, particularly during match play when intensity and physical demands peak [2, 8, 17]. However, the incidence observed in this tournament is slightly higher than reported in previous studies. A plausible explanation for this difference is the cumulative physical load inherent to a tournament setting, where several matches are played within a short time frame. Most previous studies have assessed injury incidence during individual matches rather than tournaments, and therefore may not have captured the additional fatigue and increased exposure that accumulate across multiple games. This is also reflected in the rising injury incidence as the tournament progressed. This context likely contributed to the slight increase in injury incidence observed compared with previous studies, and highlights the importance of considering tournament‐specific demands when interpreting injury patterns.

Across the six days of competition, injury incidence showed a gradual upward trend, with the lowest incidence observed on the first day and the highest on day six. This pattern aligns with previous research on other sports demonstrating that injury risk tends to increase as tournaments progress [14]. This is likely due to the combined effects of cumulative fatigue and the higher match intensity that typically develops later in competition. Although not measured, reduced or lower‐quality sleep may also contribute, as most tournament players sleep in hotels, dormitories, or on gymnasium floors alongside their peers, which can affect sleep duration and quality. Reduced sleep alone has been shown to be associated with slower reaction times, decreased performance, and generally increased injury‐related risk behaviours [27]. In combination with higher match intensity and workload, it can disrupt the balance between training load and recovery [28].

A notable finding was the small peak in injury incidence on day three. Although the overall pattern suggests a steady increase across the week, day three also saw all six injuries requiring ambulance transport and half of the injuries requiring other transportation for further medical evaluation. The reason for this spike remains uncertain. It may represent an early point at which accumulated load begins to exceed players' capacity, or it may simply reflect normal variability in injury occurrence. Nevertheless, the clustering of more severe cases on this particular day underscores the need for careful monitoring not only toward the end of a tournament, when incidence is highest, but also during the mid‐tournament phase where sudden increases can occur.

Age‐related differences in injury incidence were evident in this study, with the youngest participants showing the lowest injury rates. Players in the age groups 10 and 11 years had significantly lower injury incidences (13.9, 95% CI 7.1–20.6, and 13.4, 7.8–19.0 injuries/1000 player hours, respectively), compared with all older age categories (12–21 years; 29.5–45.7, 95% CI 25.2–51.7, injuries/1000 player hours). These findings are consistent with previous studies showing that injury incidence increases with age [2, 29]. However, most studies on youth injury incidence focus on players aged 16–18 years [25, 29], and only a few have included a broader range of age groups [8, 17].

The substantially higher rates observed between the youngest groups and players aged 12–21 years suggest that as children grow older, the game becomes faster, more physical, and more tactically complex, thereby increasing exposure to situations with greater injury potential. Notably, the 15‐year‐old group demonstrated the highest incidence of injuries, with significantly higher rates than both younger and older age groups. This pattern coincides with a developmental period characterized by rapid growth and maturation. This peak may correspond to a period in which rapid physical development, marked by Peak Heigh Velocity (PHV), is accompanied by temporary alterations in body proportions and neuromuscular control—including neuromuscular control, postural stability, and intersegmental coordination. During this phase, rapid skeletal growth may temporarily outpace the adaptation of muscles and tendons, potentially increasing mechanical stress and injury susceptibility. These transient impairments could create conditions that elevate injury risk [30, 31]. Overall, the results reinforce the importance of age‐specific injury‐prevention strategies, particularly for adolescents who appear to be at increased risk compared with younger players. Although chronological age was used in the present study, individual differences in biological maturation may influence injury risk during adolescence. Investigating injury incidence in relation to biological or pubertal age would be an interesting topic for future research.

Injury location patterns in this study were consistent with previous research, with lower‐extremity injuries being the most common, followed by wound injuries and upper‐extremity injuries (30%, 29%, and 28%, respectively) [2, 3, 32]. When stratified by sex, lower‐extremity injuries were most frequent among girls, whereas boys most commonly sustained upper‐extremity injuries. This finding has previously been reported where male youth players had a significantly higher frequency of shoulder injuries, while female youth players had a significantly higher frequency of lower body injuries [9]. Interestingly, previous research suggests that female youth handball players are at a higher risk of knee injuries during periods of increased load compared with age‐matched male players, indicating potential sex‐related differences in load tolerance [33]. In the present study, a significant association between sex and injury location was observed; however, the overall effect size was very small, indicating that the overall magnitude of these differences is limited. The rise in physical demands during a tournament may explain the patterns observed in this study. These findings highlight that while injuries can occur across the body, the lower extremities are particularly vulnerable, and that sex‐specific differences in injury prevalence underscore the importance of preventive programs tailored to the distinct risks faced by boys and girls in youth handball.

From a practical perspective, the findings of this study may contribute to improved awareness of injury risk in youth tournament settings. Such tournaments are typically characterized by a condensed competition schedule, where players may participate in several matches over a short period with limited recovery time between games. When planning future tournaments, organizers may therefore consider the potential benefits of sufficient recovery opportunities between matches and, where possible, scheduling structures that allow additional rest periods during the tournament.

In addition, the observed distribution of injuries across body locations may help identify areas that warrant particular attention during tournament participation. Increased awareness of commonly affected body regions may support targeted preventive attention by coaches and medical staff during preparation and participation in tournaments. Finally, the increased injury incidence observed during early adolescence highlights the importance of paying particular attention to players in this age range during high‐intensity competition periods such as tournaments.

## 5 Strengths and Limitations

There are several strengths of this study. It is one of the few studies regarding youth handball tournaments with the largest cohorts and players from all over the world. The prospective study design, where all injuries were recorded by medically trained staff, is a strength compared with other self‐reported or coach‐reported questionnaires. Furthermore, it includes a wide range of age groups from 10 to 21 years. However, there are limitations that need to be acknowledged. While body location was included, type of injury and severity were not included. Studies on sports injuries during games sometimes consider “absence from play,” which was not possible to control during this tournament. Although, as suggested by previous studies and in line with the purpose of this study, injury was defined as “seeking medical care”. Another possible, yet unknown, limitation is the possibility of seeking medical care elsewhere. To avoid this, the medical tents were strategically placed close to the fields. Even for more severe injuries occurring on the field, medical staff from the tournament were first on site. Thus, players potentially seeking medical care without first seeing the medical tents are considered negligible in this study.

## 6 Conclusion

During this large‐scale, six‐day youth handball tournament, the overall injury rate was 34.9 injuries per 1000 player hours. Injury incidence increased as the tournament progressed, with the highest rates on the final day and a slight mid‐tournament peak. Younger players (10–11 years) had the lowest injury rates, while 15‐year‐olds were at greatest risk. Injuries affected both upper‐ and lower‐extremities, with no major sex differences. These findings highlight the importance of age‐ and tournament‐specific injury‐prevention strategies in youth handball.

## 7 Perspectives

The large‐scale and prospective injury surveillance data presented in this study provide an important foundation for developing age‐ and tournament‐specific injury prevention strategies in youth handball. By identifying injury incidence across competition days, age groups, and anatomical locations, the findings help contextualize when and where injury risk is highest during intensive tournament play.

Future methodological improvements could include more detailed assessment of injury characteristics, particularly injury severity and recurrence, to further strengthen prevention‐oriented interpretations. Enhanced injury classification or follow‐up beyond on‐site medical care could provide additional insight into the clinical consequences of injuries.

Together, such approaches may complement large‐scale tournament surveillance and contribute to more targeted and evidence‐informed preventive efforts in youth handball.

## Funding

The authors have nothing to report.

## Ethics Statement

This study was approved by the Swedish Ethical Review authority with diary number 2023‐06647‐01.

## Conflicts of Interest

One of the authors (IL) has served as a medical doctor during the tournament in question; however, he has not received any reimbursement for this study. The other authors declare no conflicts of interest.

## Supporting information


**Table S1:** IRRs with 95% CI between all age groups. Tables S1‐S3 show injury incidence rate ratios (IRRs) between age groups (S1), day of the tournament (S2), and sex (S3). The IRRs were calculated, according to Knowles et al. [14], as the ratio of two incidence rates (injuries/1000 player hours).
**Table S2:** IRRs with 95% CI between all days in the tournament.
**Table S3:** IRR with 95% CI between sex.
**Table S4:** Adjusted standardized residual from the Chi2 examining sex differences in injury location.

## Data Availability

The data that support the findings of this study are available from the corresponding author upon reasonable request.
