# Supplementary material for: High Injury Incidence Among Youth in the World's Largest Handball Tournament: A Prospective Observational Study of 17 034 Participants
Source: Scand J Med Sci Sports. 2026 Apr 11;36(4):e70285. doi: 10.1111/sms.70285 (PMC13069875; doi:10.1111/sms.70285)
Supplement: Supplementary file 1 — Table S1: IRRs with 95% CI between all age groups. Tables S1‐S3 show injury incidence rate ratios (IRRs) between age groups (S1), day of the tournament (S2), and sex (S3). The IRRs were calculated, according to Knowles et al. [14], as the ratio of two incidence rates (injuries/1000 player hours). Table S2: IRRs with 95% CI between all days in the tournament. Table S3: IRR with 95% CI between sex. Table S4: Adjusted standardized residual from the Chi2 examining sex differences in injury location. [file SMS-36-e70285-s001.docx]

**Supplementary material 1**

Tables S1-S3 show injury incidence rate ratio (IRRs) between age groups (S1), day of the tournament (S2) and sex (S3). The IRRs were calculated, according to Knowles et al.^[[1]](#endnote-1)^, as the ratio of two incidence rates (injuries/1000 player hours).


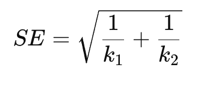


The standard error (SE) of the log (IRR) was estimated using the formula: , where k_1_ and k_2_ represent the number of injuries in the respective groups. The 95% confidence interval (CI) for the IRRs were calculated using the exponential of the log(IRR)±1.96xSE: Estimated 95% CI = exp (ln IRR ± 1.96 x SE (ln IRR)).

If the 95% CI did **not** include 1.00, the difference in injury rates was considered statistically significant. **Bold 95% CI** indicate a statistically significant difference between the groups.

**Table S1. IRRs with 95% CI between all age groups**

| **Age group** | **IRR** | **95% CI** |
| --- | --- | --- |
| 10 vs 11 | 0.97 | 0.51-1.85 |
| 10 vs 12 | 2.34 | **1.37-3.99** |
| 10 vs 13 | 2.30 | **1.37-3.86** |
| 10 vs 14 | 2.79 | **1.68-4.65** |
| 10 vs 15 | 3.30 | **1.99-5.48** |
| 10 vs 16 | 2.13 | **1.28-3.55** |
| 10 vs 18 | 3.15 | **1.89-5.25** |
| 10 vs 21 | 2.37 | **1.28-4.36** |
| 11 vs 12 | 2.41 | **1.50-3.86** |
| 11 vs 13 | 2.37 | **1.51-3.72** |
| 11 vs 14 | 2.88 | **1.85-4.47** |
| 11 vs 15 | 3.40 | **2.20-5.27** |
| 11 vs 16 | 2.20 | **1.41-3.42** |
| 11 vs 18 | 3.25 | **2.09-5.06** |
| 11 vs 21 | 2.44 | **1.40-4.25** |
| 12 vs 13 | 0.98 | 0.75-1.30 |
| 12 vs 14 | 1.20 | 0.92-1.55 |
| 12 vs 15 | 1.41 | **1.10-1.82** |
| 12 vs 16 | 0.91 | 0.70-1.18 |
| 12 vs 18 | 1.35 | **1.04-1.75** |
| 12 vs 21 | 1.01 | 0.66-1.55 |
| 13 vs 14 | 1.22 | 0.98-1.51 |
| 13 vs 15 | 1.44 | **1.16-1.78** |
| 13 vs 16 | 0.93 | 0.74-1.16 |
| 13 vs 18 | 1.37 | **1.10-1.71** |
| 13 vs 21 | 1.03 | 0.69-1.54 |
| 14 vs 15 | 1.18 | 0.98-1.43 |
| 14 vs 16 | 0.76 | **0.62-0.93** |
| 14 vs 18 | 1.13 | 0.92-1.38 |
| 14 vs 21 | 0.85 | 0.57-1.25 |
| 15 vs 16 | 0.80 | **0.65-0.97** |
| 15 vs 18 | 1.18 | 0.97-1.43 |
| 15 vs 21 | 0.88 | 0.60-1.30 |
| 16 vs 18 | 1.03 | 0.84-1.27 |
| 16 vs 21 | 0.78 | 0.52-1.15 |
| 18 vs 21 | 0.75 | 0.51-1.11 |

**Table S2. IRRs with 95% CI between all days in the tournament**

| **Day in the tournament** | **IRR** | **95% CI** |
| --- | --- | --- |
| 1 vs 2 | 1.04 | 0.38-2.80 |
| 1 vs 3 | 1.92 | 0.72-5.15 |
| 1 vs 4 | 1.47 | 0.55-3.95 |
| 1 vs 5 | 2.02 | 0.75-5.41 |
| 1 vs 6 | 2.18 | 0.79-6.01 |
| 2 vs 3 | 1.85 | **1.53-2.24** |
| 2 vs 4 | 1.42 | **1.16-1.73** |
| 2 vs 5 | 1.95 | **1.61-2.35** |
| 2 vs 6 | 2.10 | **1.54-2.86** |
| 3 vs 4 | 0.77 | **0.65-0.91** |
| 3 vs 5 | 1.05 | 0.90-1.23 |
| 3 vs 6 | 1.13 | 0.85-1.51 |
| 4 vs 5 | 1.00 | 0.84-1.18 |
| 4 vs 6 | 1.08 | 0.80-1.45 |
| 5 vs 6 | 1.00 | 0.75-1.44 |

**Table S3. IRR with 95% CI between sex**

| **Sex** | **IRR** | **95% CI** |
| --- | --- | --- |
| Boys vs girl | 0.96 | 0.85-1.09 |

**Table S4. Adjusted standardized residual from**

**the Chi^2^ examining sex differences in injury location**

|  | **Girls** | **Boys** |
| --- | --- | --- |
| Face | -0.8 | 0.8 |
| Head | **2.8** | **-2.8** |
| Neck/back | 0.3 | -0.3 |
| Upper extremity | **-2.5** | **2.5** |
| Lower extremity | 0.1 | -0.1 |
| Multi | 1.9 | -1.9 |
| Wound | 0.5 | -0.5 |
| Abdomen | 0.1 | -0.1 |
| Other | 1.8 | -1.8 |

1. Knowles SB, Marshall SW, Guskiewicz KM. Issues in estimating risks and rates in sports injury research. *J Athl Train.* 2006;41(2):207-215 [↑](#endnote-ref-1)
